# Metabolic enzyme ACSL3 is a prognostic biomarker and correlates with anticancer effectiveness of statins in non‐small cell lung cancer

**DOI:** 10.1002/1878-0261.12816

**Published:** 2020-10-30

**Authors:** Lara Paula Fernández, María Merino, Gonzalo Colmenarejo, Juan Moreno‐Rubio, Ruth Sánchez‐Martínez, Adriana Quijada‐Freire, Marta Gómez de Cedrón, Guillermo Reglero, Enrique Casado, María Sereno, Ana Ramírez de Molina

**Affiliations:** ^1^ Molecular Oncology Group IMDEA Food Institute CEI UAM + CSIC Madrid Spain; ^2^ Medical Oncology Department Infanta Sofía University Hospital San Sebastián de los Reyes Madrid Spain; ^3^ Biostatistics and Bioinformatics Unit IMDEA Food Institute CEI UAM+CSIC Madrid Spain

**Keywords:** cholesterol, fatty acids, lipid metabolism, non‐small cell lung cancer, prognosis, statins

## Abstract

Lung cancer is one of the most common cancers, still characterized by high mortality rates. As lipid metabolism contributes to cancer metabolic reprogramming, several lipid metabolism genes are considered prognostic biomarkers of cancer. Statins are a class of lipid‐lowering compounds used in treatment of cardiovascular disease that are currently studied for their antitumor effects. However, their exact mechanism of action and specific conditions in which they should be administered remains unclear. Here, we found that simvastatin treatment effectively promoted antiproliferative effects and modulated lipid metabolism‐related pathways in non‐small cell lung cancer (NSCLC) cells and that the antiproliferative effects of statins were potentiated by overexpression of acyl‐CoA synthetase long‐chain family member 3 (ACSL3). Moreover, ACSL3 overexpression was associated with worse clinical outcome in patients with high‐grade NSCLC. Finally, we found that patients with high expression levels of ACSL3 displayed a clinical benefit of statins treatment. Therefore, our study highlights ACSL3 as a prognostic biomarker for NSCLC, useful to select patients who would obtain a clinical benefit from statin administration.

## Graphical abstract

Overexpression of the enzyme ACSL3 is an unfavorable prognostic marker in non‐small cell lung cancer (NSCLC). Moreover, overexpression of ACSL3 increases cell proliferation, migration, and invasion, altering the metabolic properties of cancer cells. Administration of statins causes antiproliferative effects and, importantly, patients with high expression levels of ACSL3 following statin treatment display better prognosis than those not treated with statins.

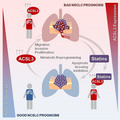

**Abbreviations**
3‐HMGCR: 3‐hydroxy‐3‐methylglutaryl‐coenzyme A reductase
95% CI: 95% confidence intervals
ACSL3: acyl‐CoA synthetase long‐chain family member 3
ACSLs: long‐chain acyl‐CoA synthetases
ALP: alkaline phosphatase
APOA1: apolipoprotein A1
ATCC: American Type Culture Collection
CASP9: caspase 9
ECAR: extracellular acidification rate
ECOG: Eastern Cooperative Oncology Group
EMT: epithelial‐to‐mesenchymal transition
ER: endoplasmic reticulum
FAs: fatty acids
FFPE: formalin‐fixed, paraffin‐embedded
GTEx: genotype‐tissue expression
HR: Hazard ratio
IC50: half‐maximal inhibitory concentration
LDH: lactate dehydrogenase
MTT: 3‐(4,5‐dimethylthiazol‐2‐yl)‐2,5‐diphenyltetrazolium
NID1: nidogen 1
No ORF: no open reading frame
NSCLC: non‐small cell lung cancer
OCR: oxygen consumption rate
OS: overall survival
PGE2: prostaglandins E2
RETN: resistin
TCGA: The Cancer Genome Atlas
TMA: tumor tissue microarray

## 1 Introduction

Lung neoplasia is the leading cause of cancer death in both sexes, accounting for almost 20% of the total cancer deceases [1]. Non‐small cell lung cancer (NSCLC) is the most frequent subtype and represents about 85% of all lung tumors [2]. Genetic and environmental factors have been associated with the development of NSCLC, with cigarette smoking being the most important modifiable risk factor. Accordingly, it is estimated that 70% of all NSCLC tumors arise in smokers [3].

Unfortunately, NSCLC is frequently diagnosed when advanced‐stage disease is present [4, 5]. The 5‐year overall survival rate for NSCLC remains poor, from 68% in patients with stage IB disease to 0–10% in patients with stage IVA‐IVB disease [6]. During the last decade, a significant improvement in the clinical outcome of patients has been reached, essentially due to the integration of targeted therapies and immunotherapy in genetically selected subpopulations of patients [7]. However, only few NSCLC patients will achieve long‐term survival, and therefore, a deeper understanding of NSCLC molecular biology coupled with high‐quality biomarkers of the disease is urgently needed.

Deregulation of cellular metabolism is a hallmark of cancer [8]. Tumor cells adapt their metabolic status to satisfy their novel requirements of growth, proliferation, and survival. Together with the Warburg effect, lipid metabolism contributes to cancer metabolic reprogramming [9]. Moreover, lipid metabolism genes have a promising role as prognostic biomarkers of cancer. Their deregulated expression has been associated with cancer recurrences and survival [10, 11]. Nevertheless, the exact mechanism of the regulation and the biological consequences of lipid metabolism in NSCLC have not been elucidated yet.

Fatty acids (FAs) are indispensable components of cellular membranes, and they are essential for post‐translational protein modifications; moreover, they are energy generators and contribute to maintain redox homeostasis through β‐oxidation [12, 13, 14]. The enzymatic family of long‐chain acyl‐CoA synthetases (ACSLs) transform free long‐chain FAs into fatty acyl‐CoA esters, which are used for both lipid synthesis and β‐oxidation [15]. ACSL enzymes are ubiquitously expressed, although they differ in tissue expression and in their subcellular localization. For instance, ACSL3 is basically confined to endoplasmic reticulum (ER) and lipid droplets and ACSL4 is localized in peroxisomes and ER, while ACSL1, ACSL5, and ACSL6 are found in mitochondria, plasma membrane, and cytoplasm [16, 17]. As a matter of fact, ACSLs enzymes are involved in the metabolic reprogramming of tumor cells. For example, ACSLs stimulate colon cancer progression increasing the metabolic activity as well as the invasive and migratory properties of malignant cells [18]. Besides, the pharmacologic inhibition of ACSLs promotes apoptosis in a TP53‐deficient cancer context [19, 20].

Other fundamental structural component of lipid membranes is cholesterol, that is also essential for cellular proliferation [21]. Tumor cells show a powerful fatty acid and cholesterol avidity, which can be satisfied by increasing the uptake of exogenous lipids and/or lipoproteins or by activating *de novo* lipogenesis and cholesterogenesis [9]. Increased serum cholesterol levels have been associated with higher risk of cancer [22]. Statins are 3‐hydroxy‐3‐methylglutaryl‐coenzyme A reductase (3‐HMGCR) inhibitors, and they are extensively used in the treatment of hypercholesterolemia [23]. Besides, *in vitro* and *in vivo* experimental data suggest that statins may also exert oncoprotective effects [24, 25, 26]. They have been implicated in the regulation of inflammation, immunomodulation, and angiogenesis [27] too. Particularly, in lung cancer cells, statins induce apoptosis [28], and in preclinical models, statins suppress tumor growth [29]. It has been reported that the long‐term use of statins in patients with lung cancer and dyslipidemia decreases mortality [30]. However, not always consistent results are found in the epidemiological and clinical studies that explored the protective role of statins in lung cancer [30, 31, 32]. Notably, the effect of the use of statins in NSCLC patients, taking into account their lipid metabolic reprogramming, remains unexplored.

The prognostic value of lipid metabolism genes in high‐grade NSCLC patients is evaluated for the first time in this work. Our goal is to define putative lipid metabolism‐related biomarkers of survival and to analyze their functional consequences and mechanism of action. Moreover, we deepen into the effect of statins in specific metabolic subgroups of patients, providing novel precision medicine strategies for specific subgroups of NSCLC patients.

## 2 Materials and methods

### 2.1 Patient’s selection

A total number of 90 formalin‐fixed, paraffin‐embedded (FFPE) samples were obtained from NSCLC patients of the Medical Oncology Service of Infanta Sofía University Hospital (San Sebastián de los Reyes, Madrid, Spain). Advanced NSCLC patients enrolled from through November 1, 2008, to December 31, 2015, were included in this analysis. All cases were of high stage (stages III–IV, and they were treated following a standardized protocol. All FFPE samples were studied by an anatomic pathologist confirming > 80% of tumoral cells in each case. Clinical and pathological data were collected from medical reports (see Table 1). This study was approved by the local ethical committee, and all patients signed the consent inform document. The study methodologies conformed to the standards set by the Declaration of Helsinki. Overall survival (OS) was defined from the date of diagnosis to the date of patient death. We also used population’s data extracted from 984 patients from The Cancer Genome Atlas (TCGA) public dataset which characteristics were summarized in Table S1.

**Table 1:** Clinical characteristics of non‐small cell lung cancer patients from initial set.

Characteristics	HR (95% CI)	*P*‐value OS	N (%)
Non‐small cell lung cancer (NSCLC) patients			90 (100)
Overall survival			
N° Exitus			77 (85.6)
Age at diagnosis (years)	1.02 (1.00–1.04)	0.1	
Mean			64.13
Median			63.5
Age range			40–91
Under 50			6 (6.7)
50–70			59 (65.6)
Over 70			25 (27.8)
Gender	0.86 (0.50–1.47)	0.6	
Female			22 (24.4)
Male			68 (75.6)
Histology	0.56 (0.32–0.97)	0.03	
Adenocarcinoma			68 (75.6)
Squamous			22 (24.4)
Stage	3.27 (1.86–5.76)	8.64 × 10^−06^	
III			25 (27.8)
IIIA			9 (10.0)
IIIB			16 (17.8)
IV			65 (72.2)
ECOG performance status	2.78 (2.03–3.82)	1.03 × 10^−09^	
0			16 (17.8)
1			46 (51.1)
2			22 (24.4)
3			3 (3.3)
4			2 (2.2)
Smoking history	1.45 (1.00–2.11)	0.05	
Nonsmoker			12 (13.3)
Current			47 (52.2)
Ex smoker			31 (34.4)
Metastasis	2.54 (1.55–4.17)	1.37 × 10^−04^	
No			35 (38.9)
Yes			55 (61.1)
Weight loss	1.04 (0.58–1.86)	0.9	
No			75 (83.3)
Yes			15 (16.7)
BMI (kg·m^−2^)	0.96 (0.89–1.03)	0.2	
Mean			25.7
< 25			33 (36.7)
25–30			35 (38.9)
≥ 30			12 (13.3)
Total cholesterol	1.00 (0.99–1.00)	0.5	
< 200 mg·dL^−1^			52 (57.8)
≥ 200 mg·dL^−1^			10 (11.1)
HDL	1.01 (1.00–1.03)	0.2	
< 35 mg·dL^−1^			10 (11.1)
≥ 35 mg·dL^−1^			44 (48.9)
LDL	1.00 (0.99–1.01)	0.8	
< 130 mg·dL^−1^			47 (52.2)
≥ 130 mg·dL^−1^			7 (7.8)
Statins treatment	0.77 (0.45–1.33)	0.3	
No			66 (73.3)
Yes			24 (26.7)
Diabetes mellitus	1.65 (0.93–2.93)	0.1	
No			73 (81.1)
Yes			17 (18.9)
Metformin treatment	1.71 (0.81–3.61)	0.2	
No			81 (90)
Yes			9 (10)
EGFR mutational status			
Negative			84 (93.3)
Positive			6 (6.7)
ALK status			
Negative			90 (100)
Positive			0 (0)

### 2.2 Gene expression analysis

We employed RNeasy Mini Kit or RNeasy FFPE Kit (Qiagen, Germantown, MD, USA) to get total RNA from NSCLC cancer cells or from FFPE tumor samples previously deparaffinated, respectively. One μg of total RNA was reverse‐transcribed by High Capacity cDNA Archive Kit (Applied Biosystems, Waltham, MA, USA) for 2 h at 37 °C.

VeriQuest SYBR Green qPCR Master Mix (Affymetrix, Santa Clara, CA, USA) was applied for gene expression analysis of NSCLC cells as previously described [33, 34] (see oligos used in Table S2). In clinical samples, a Taq‐Man Low Density Array (Applied Biosystems) was specially designed for this experiment and was formed of 22 lipid metabolism‐related genes (Table 2).

**Table 2:** Lipid metabolism‐related genes analyzed in this study.

Metabolic pathway	Gene symbol	Gene description
Adipocytes, obesity, type II diabetes	*RETN*	Resistin
Adipocytokine signaling and immune homeostasis	*ADIPOR1*	Adiponectin receptor protein 1
Cholesterol transport	*ABCA1*	ATP‐binding cassette, subfamily A (ABC1), member 1
	*APOC1*	Apolipoprotein C1
	*APOC2*	Apolipoprotein C2
	*APOD*	Apolipoprotein D
Fatty acid biosynthesis	*SCD*	Stearoyl‐CoA desaturase (delta‐9‐desaturase)
Fatty acid β‐oxidation	*ACADM*	Acyl‐Coenzyme A dehydrogenase, C‐4 to C‐12 straight chain
	*HMGCL*	3‐Hydroxymethyl‐3‐methylglutaryl‐Coenzyme A lyase
	*HMGCS2*	3‐Hydroxy‐3‐methylglutaryl‐Coenzyme A synthase 2 (mitochondrial)
	*PPA1*	Pyrophosphatase (inorganic) 1
Lipid metabolism in peroxisomes	*ACSL1*	Acyl‐CoA synthetase long‐chain family member 1
	*ACSL3*	Acyl‐CoA synthetase long‐chain family member 3
	*ACSL4*	Acyl‐CoA synthetase long‐chain family member 4
	*ACSL5*	Acyl‐CoA synthetase long‐chain family member 5
	*ACSL6*	Acyl‐CoA synthetase long‐chain family member 6
	*AMACR*	Alpha‐methylacyl‐CoA racemase
	*HSD17B4*	Hydroxysteroid (17‐beta) dehydrogenase 4
O‐linked glycosylation	*GCNT3*	Mucin‐type core 2 1,6N‐acetylglucosaminyltransferase
PPAR signaling	*PLIN1*	Perilipin 1
Receptors and basement membrane glycoproteins	*NID1*	Nidogen 1
Regulation of the hepatocyte growth factor (HGF)	*ST14*	Suppression of tumorigenicity 14 (colon carcinoma)

These 22 genes within all genes present in pathways related to lipid metabolism were selected due to their role as regulators of cell metabolism, key steps of interconnection among lipid pathways, or their reported role in biological processes associated with cancer [10]. Gene expression assays were performed in a HT–7900 Fast Real‐time PCR. The *B2M* gene expression was used as endogenous control. rt‐statminer software (Integromics^®^ Inc., Madison, WI, USA) was used to detect and determine the quality control and differential expression analyses of data [10].

### 2.3 Cell culture, treatments, and stable cell lines generation

A panel of NSCLC cells (CRL 5803, CRL 5872, CRL 5875, CRL 5877, and CRL 5908) and HEK‐293T cells was obtained from American Type Culture Collection (ATCC, Middlesex, UK). ATCC performs cell line authentication through STR profiling and mycoplasma contamination testing. Frozen aliquots were stored, and cells were passaged in the laboratory for fewer than 6 months after resuscitation [35]. All cell lines were cultured and maintained under manufacture’s conditions.

Generation of ACSL3 stable overexpression cellular models was performed using lentiviral systems as previously described [18]. HEK‐293T cells were transfected using Lipofectamine 2000 (Life Technologies, Thermo Fisher Scientific, Waltham, MA, USA) with lentiviral vectors expressing ACSL3/No ORF empty vector (DNA 2.0, Newark, CA, USA) along with a set of packaging plasmids (Addgene, Cambridge, MA, USA) [18, 35].

Cells were treated with vehicle or simvastatin or pravastatin (Sigma‐Aldrich, St. Louis, MO, USA) at various concentrations. Cell viability was determined by the 3‐(4,5‐dimethylthiazol‐2‐yl)‐2,5‐diphenyltetrazolium (MTT) assay as previously described [18].

### 2.4 Antibodies and western blotting

We used primary antibodies against ACSL3 (Invitrogen‐Thermo Fisher Scientific, Waltham, MA, USA, PA5‐42883) and β‐Actin (Sigma‐Aldrich, A1978). The following secondary antibodies were used: Horseradish peroxidase‐conjugated antibodies anti‐mouse and anti‐rabbit (Merck‐Millipore, Darmstadt, Germany). Cells were lysed, and proteins were separated by SDS–polyacrylamide gel electrophoresis and transferred into a nitrocellulose membrane (Bio‐Rad, Hercules, California, USA) as previously described [18, 35].

### 2.5 Proliferation, migration and invasion assays

Cell proliferation was measured in real time using the xCELLigence™ system (ACEA Biosciences, San Diego, CA, USA) according to manufacturer’s protocols. Real‐time monitoring of proliferation was performed for 5 days in 30‐min intervals [35].

A density of 100 000 cells per well was seeded in serum‐free medium into inserts of a BD migration and MatrigelTM invasion chamber (BD Biosciences, San Jose, CA, USA). Migrative and invasive cells were measured as previously described [18, 35].

### 2.6 Extracellular flux analysis of the acidification rate (ECAR) and the oxygen consumption rate (OCR)

Aerobic glycolysis (Cell GlycoStress Test) and mitochondrial oxidative respiration (Cell MitoStress Test) were examined with the XFe96 Cell Bionalyzer (Seahorse Biosciences, North Billerica, MA, USA, XFe96). Optimal cell density and drugs titration were determined as previously described [36]. The requirements of the cells for aerobic glycolysis and oxidative phosphorylation were screened after the injection of several modulators of both bioenergetic pathways. We monitored mitochondrial respiration and glycolytic function using XF Cell Mito Stress Test kit and XF Glycolysis Stress kit according to manufacturer instructions (Seahorse Biosciences). Briefly, for GlycoStress assay, 20 000 cells were seeded in an XFe96 well plate and maintained in DMEM 10% FBS to allow the cells to attach [36]. Then, the culture medium was changed to 2 mm glutamine without glucose in XFe DMEM (5 mm Hepes) to starve the cells for 1 h. First basal extracellular acidification rate (ECAR) was determined (1–3 measurements). Glucose (10 mm final concentration) was injected (4–6 measurements) to measure glycolysis (i.e., the increased ECAR from basal starved situation after glucose addition). This indicates the competence of the cells to employ glucose. Next, maximal glycolytic capacity was assayed (7–9 measurements) after the injection of oligomycin, which blocks the ATP production from the oxidative mitochondrial respiration. Finally, a third injection with 50 mm DG final concentration was done to shut down aerobic glycolysis (10–12 measurements) [36]. For MitoStress assay, 40 000 cells were seeded in an XFe96 well plate, and cells were maintained in DMEM 10% FBS to allow the cells to attach. Then, the medium was changed to 10 mm glucose, 2 mm glutamine, and 1 mm pyruvate in XFe DMEM (5 mm Hepes), and cells were incubated for 1 h at 37 °C without CO^2^. Three different modulators of the mitochondrial respiration were sequentially introduced. After basal oxygen consumption rate (OCR) determination (1–3 measurements), oligomycin (1 µm), which inhibits ATPase, was added to determine the amount of oxygen dedicated to ATP production by mitochondria (3–6 measurements) [36]. To determine the maximal respiration rate or spare respiratory capacity, FCCP (carbonyl cyanide‐4‐(trifluoromethoxy)phenylhydrazone) was injected (0.4 µm) to free the gradient of H^+^ from the mitochondrial intermembrane space (7–9 measurements) and thus to activate maximal respiration. Finally, antimycin A and rotenone (0.5 µm) were added to completely inhibit the mitochondrial respiration (10–12 measurements) [36]. At least 6 replicates per condition were done for each experiment.

### 2.7 Statistical analysis

The primary endpoint of the study was the relationship between gene expression and overall survival (OS). Gene expression (Q) in tumor samples was quantified with the 2–Δ*C*_t_ method as previously described [35]. Violin plots were used to display and analyze the distributions of gene expression. The Kaplan–Meier method was used to estimate overall survival curves, and univariate Cox regression analysis was used to test the association between overall survival and clinical variables. Multivariable Cox regression models were derived to estimate the hazard ratio of gene expression and adjusting for potential confounding factors: age, sex, stage, ECOG, and smoking status. In order to minimize the effect of putative outliers and influential points, instead of a continuous gene expression variable a binarized version of it was used by determining a cut‐point based on maximally selected rank statistics and using Log‐rank scores [37]. The absence of influential observations or outliers was afterward verified through the analysis of the residuals deviance and the changes in the standardized coefficients upon deletion of each observation [37]. In addition, proportional hazards assumption in the Cox models was verified both through tests as described by Grambsch *et al*. [38] and by inspection of the Schoenfeld scaled residuals against time. Hazard ratios (HR) and their 95% confidence intervals (95% CI) and *P*‐values were calculated from the Cox regression modes. Statistical significance was defined as *P*‐value < 0.05 with bilateral tests. Bonferroni method was applied for multiple test correction of the *P*‐values. The statistical analyses were performed using the R statistical software version 3.6.1 (www.r‐project.org).

Student’s *t*‐test was employed to assay statistically significant differences between sets. All reported *P*‐values were two‐sided. Statistical significance was defined as *P* < 0.05.

## 3 Results

### 3.1 Metabolic reprogramming in NSCLC tumors is a reliable prognostic marker of survival

Altered lipid metabolism has been found in many types of cancer, and consequently, lipid metabolism genes could constitute a prognostic and therapeutic tool in these tumors [10, 11, 39]. In order to explore the putative prognostic value of lipid metabolism genes in NSCLC patients, we performed survival analysis based on gene expression in a series of 90 high‐grade NSCLC tumor samples. The final dataset included 25 and 65 samples of stages III and IV, respectively. Median age at diagnosis was 63.5 years (range 40–91), and median overall survival (OS) was 7 months (range 0–62). Only 13.3% of patients had never been smokers. Sample characteristics are described in Table 1. The estimated HRs for OS of various phenotypic characteristics based on univariate Cox models are also shown. As expected, tumor stage, ECOG (Eastern Cooperative Oncology Group) performance status, and presence of metastasis were associated with higher risk of death, with HRs of 3.27 (95% CI: 1.86–5.76), 2.78 (95% CI: 2.03–3.82), and 2.54 (95% CI: 1.55–4.17), respectively.

A transcriptomic panel of lipid metabolism genes was assayed in NSCLC samples. Gene selection was made according to our previous analysis in two other types of cancer: colorectal and epithelial ovarian cancer [10, 11, 35]. Genes were chosen due to their previous described functionality as well as according to their predicted expression in lung tissue (https://www.proteinatlas.org/). Finally, 22 lipid metabolism genes were included in the panel which is shown in Table 2.

**Fig. 1:**
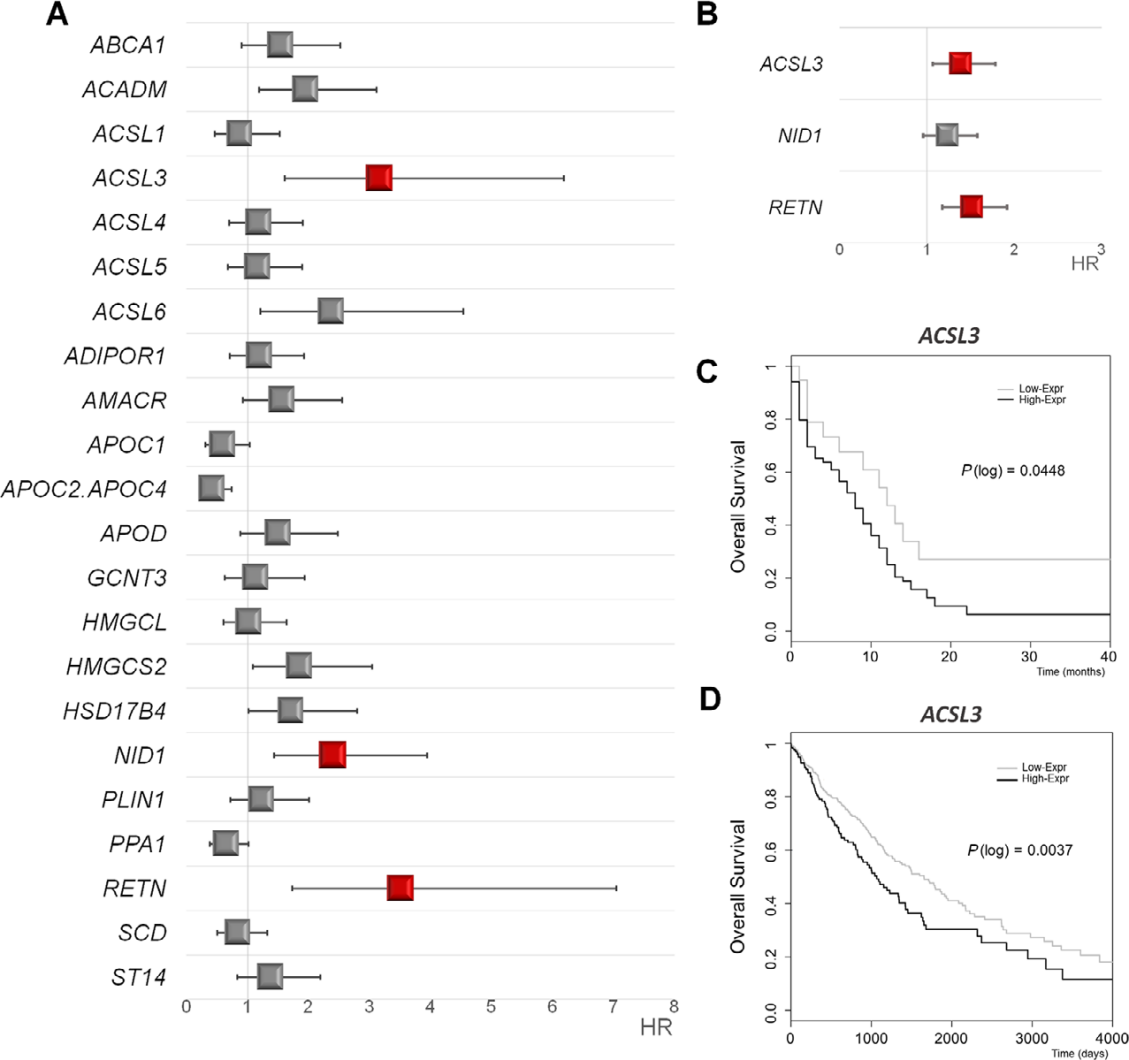
Lipid metabolism gene associations with NSCLC survival: Results from Initial and Validation sets. (A) Forest plot showing the hazard ratio (HR) and 95% confidence interval (CI) estimates for overall survival (OS) of lipid metabolism gene expression in 90 non‐small cell lung cancer (NSCLC) patients from our dataset. (B) Forest plot showing the hazard ratio (HR) and 95% confidence interval (CI) estimates for overall survival (OS) of *ACSL3*, *NID1,* and *RETN* expression in 984 NSCLC patients from The Cancer Genome Atlas (TCGA) study (validation set). (C) Kaplan–Meier plots for *ACSL3* expression in 90 NSCLC patients from Initial set (Low‐Expr: low *ACSL3* gene expression group, High‐Expr: high *ACSL3* gene expression group, *P*(log): *P*‐value log‐rank). (D) Kaplan–Meier plots for *ACSL3* expression in 984 NSCLC patients from TCGA study (validation set) (Low‐Expr: low *ACSL3* gene expression group, High‐Expr: high *ACSL3* gene expression group, *P*(log): *P*‐value log‐rank).

After adjusted analysis for putative confounder variables (age, sex, stage, ECOG, histology, and smoking status), we found statistically significant associations with OS for three genes: acyl‐CoA synthetase long‐chain family member 3 (*ACSL3*), a fatty acid activating isoform essentially located in the ER and lipid droplets; Nidogen 1 (*NID1*), a basement membrane glycoprotein; and Resistin (*RETN*), an adipokine. Results are shown in Fig. 1A. Accordingly, individual risks of decease hazard ratios for *ACSL3*, *NID1,* and *RETN* high‐expressing NSCLC patients were 2.93 (95% CI: 1.5–5.73), *P* = 0.011; 2.2 (95% CI: 1.32–3.64), *P* = 0.04, and 3.23 (95% CI: 1.61–6.49), *P* = 0.004, respectively. Violin plots showing distributions of *ACSL3*, *NID1,* and *RETN* gene expression are represented in Fig S1.

Next, we assessed for associations of *ACSL3*, *NID1,* and *RETN* expression with the clinical and/or tumoral characteristics in NSCLC patients. We only detected that *ACSL3* expression was marginally associated with alkaline phosphatase (ALP) and lactate dehydrogenase (LDH) serum levels (OR = 10.43 (95% CI: −0.4 to 21.26), *P* = 0.06 and OR = 37.79 (95% CI: −4.44 to 80.02), *P* = 0.08, respectively).

To validate these results, we performed a parallel analysis based on *ACSL3*, *NID1,* and *RETN* expression in larger series of NSCLC samples from The Cancer Genome Atlas (TCGA) study, a publicly available gene expression dataset (https://www.cancer.gov/tcga). The analysis included a total number of 984 NSCLC patients. *ACSL3* and *RETN* expression had an overall survival risk effect, and the adjusted HR was significantly higher than 1 (1.38 (95% CI: 1.07–1.79), *P* = 0.016 and 1.51 (95% CI: 1.18–1.92), *P* = 0.001, respectively) (Fig 1B).

Then, we also checked the potential value of *ACSL3* and *RETN* as NSCLC prognostic biomarkers using The Human Protein Atlas website together with The Pathology Atlas online tool (https://www.proteinatlas.org/humanproteome/pathology), that contains mRNA data from TCGA study and protein expression data from different forms of human cancer [40, 41, 42]. Lung cancer tissues showed moderate‐to‐strong ACSL3 cytoplasmic immunoreactivity. Moreover, *ACSL3* mRNA expression scrutiny displayed analogous results to our previous analysis with a clear association between high *ACSL3* expression and poor patient’s survival (*P* score = 0.0008, *n* = 994) (https://www.proteinatlas.org/ENSG00000123983-ACSL3/pathology). All these data reliably validate our findings that upregulation of *ACSL3* is associated with low survival in NSCLC patients.

Finally, as expected, Kaplan–Meier plots for OS of *ACSL3* showed an association between high gene expression and poor clinical outcome, both in the initial group (Fig. 1C) and in TCGA validation dataset (Fig. 1D). Consequently, *ACSL3* overexpression could be considered as an unfavorable prognostic factor in NSCLC.

### 3.2 Overexpression of ACSL3 enzyme increases cell proliferation, migration, and invasion altering metabolic properties of NSCLC cells

In order to explore the role of ACSL3 as a putative NSCLC prognostic factor, we generated NSCLC cell lines overexpressing ACSL3. Firstly, we analyzed mRNA and protein expression levels of *ACSL3* in a panel of several NSCLC cell lines. Results are shown in Fig S2. None of the NSCLC cell lines analyzed expressed detectable levels of ACSL3 protein. By contrast, we were able to distinguish various levels of *ACSL3* mRNA expression among cell lines. We validated our results using data from the Cancer Cell Line Encyclopedia [43] https://portals.broadinstitute.org/ccle/about) and COSMIC (https://cancer.sanger.ac.uk/cosmic) databases. Taken all data together, for stably overexpression of ACSL3, we decided to use the two cell lines with the lowest *ACSL3* endogenous expression levels: CRL 5803 and CRL 5908. Overexpression of *ACSL3* in both cell lines was confirmed by western blot and by qPCR (mRNA) (Fig S2D,F).

The enhanced cell proliferation, migration, and invasion are three essential characteristics for cancer progression and metastasis formation. Additionally, cancer cells reprogram their metabolic properties to facilitate cell survival and proliferation. To elucidate how ACSL3 overexpression affects the carcinogenic properties of NSCLC cells, we analyzed cell growth, migration, and invasion.

**Fig. 2:**
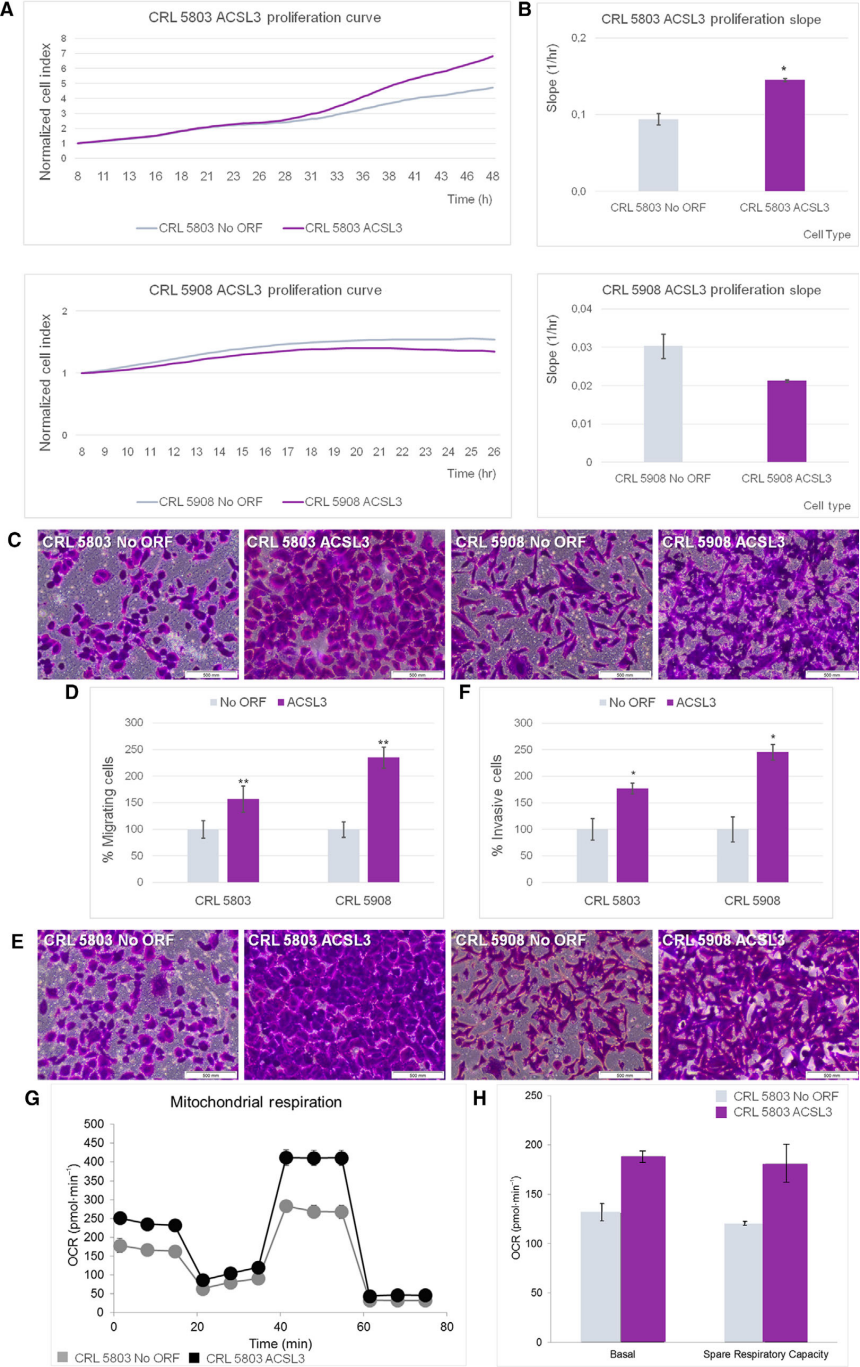
ACSL3 overexpression increases cell proliferation, migration, and invasion altering metabolic properties of NSCLC cells. (A) xCELLigence proliferation assays of No ORF and ACSL3 in CRL 5803 and CRL 5908 cells. Complete growth curves are represented from 8 to 48 h in CRL 5803 cells and from 8 to 26 h in CRL 5908 cells. Results were represented as 8‐h normalized cell index value. Data are expressed as mean ± SEM of three independent experiments each performed in triplicate. (B) Rate of cell proliferation determined by measuring the slope of the proliferation line between the 8‐ and 48‐h interval for CRL 5803 cells and between the 8‐ and 26‐h interval for CRL 5908 cells. Results were shown as 8‐h normalized cell index value. Data are expressed as mean ± SEM of three independent experiments each performed in triplicate. Student’s *t*‐test was used to evaluate statistically significant differences (*P < 0.05). (C) Boyden chamber migration transwell experiment of No ORF and ACSL3 in CRL 5803 and CRL 5908 cells. After 48 h, cells were fixed and stained with crystal violet and measured under an optical microscope. Pictures were taken employing an Olympus CKX41 microscope (Olympus, Tokyo, Japan), with a 20X LCAch objective and recorder using analysis getIT software (Olympus). Scale bars 500 µm. (D) Percentage of migrating cells counted under an optical microscope. Data are shown as mean ± SEM of three independent experiments each performed in triplicate. Student’s t‐test was used to evaluate statistically significant differences (**P < 0.01). (E) Boyden chamber transwell analysis of NoORF and ACSL3 in CRL 5803 and CRL 5908 cells invasion through Matrigel. After 48 h, cells were fixed and stained with crystal violet and measured under an optical microscope. Pictures were taken employing an Olympus CKX41 microscope (Olympus), with a 20X LCAch objective and recorder using analysis getit software (Olympus). Scale bars 500 µm. (F) Percentage of invasive cells counted under an optical microscope. Data are expressed as mean ± SEM of three independent assays each performed in triplicate. Student’s *t*‐test was used to evaluate statistically significant differences (*P < 0.05). (G) Oxygen consumption rate (OCR) of NoORF and GCNT3 CRL 5803 cells. Bioenergetics parameters were acquired by injecting 2 µm Oligomycin to inhibit ATP‐linked OCR, 0.2 µm FCCP to uncouple mitochondria for maximal OCR, and 0.5 µm Rotenone/Antimycin A (Rot/AA) to shut down mitochondrial respiration. We show OCR measurements over time for cells stably expressing NoORF or ACSL3. We illustrate representative experiments of 6 measures (*n* = 3). (E) Quantification of basal respiration (oxygen consumption used to meet cellular ATP demand, calculated by subtracting non‐mitochondrial OCR obtained upon Rot/AA injection) and spare respiratory capacity (capability to respond to an energetic demand, calculated as the difference between maximal and basal OCR) of NoORF and GCNT3 CRL 5803 cells. We illustrate representative experiments of 6 measures (*n* = 3).

We used the xCELLigence™ system to assay cell proliferation and cell viability in real time (Fig 2A,B). We found that ACSL3 overexpression increased cell growth in CRL 5803 cells (54.61% of increase in the growth curve slope (1/h), *P*‐value = 0.03 compared to the control No ORF CRL 5803). These changes were not observed in CRL 5908 cells. Then, we analyzed the migration capacities of ACSL3 overexpressing cells. We quantified cell migration starting from a serum‐free environment to exclude proliferation differences (Fig 2C,D). Significantly, up to 57.15% more cells were found to migrate in ACSL3 CRL 5803 compared to the No ORF CRL 5803 control cells, and 135.36% more cells migrated in the ACSL3 CRL 5908 condition compared to the No ORF CRL 5803 control cells (*P*‐values = 0.0075 and 0.0076, respectively). We also measured the invasive properties of ACSL3 overexpressing cells compared to their controls (Fig 2E,F). Both ACSL3 CRL 5803 and ACSL3 CRL 5908 displayed increased invasion through Matrigel™ when compared to their control cells (increases of 77.66%, *P* = 0.04 and 145.44%, *P* = 0.03, respectively).

We next analyzed the expression of a panel of epithelial‐to‐mesenchymal transition (EMT)‐associated genes. We tested *E‐cadherin*, *K18,* and *Na+/K + ATPase β1* as epithelial markers and *β‐catenin*, *N‐cadherin*, *Slug,* and *Vimentin* as mesenchymal markers (Fig S3). A decrease in the *E‐cadherin* expression levels was found in both ACSL3 overexpressing cells compared to their controls, although this reduction was only statistically significant in the ACSL3 CRL 5908 (*P* = 0.004). Also in ACSL3 CRL 5908 cells, we detected lower levels of *K18* than in control cells (*P* = 0.02). This is in agreement with a loss of epithelial characteristics and gain of an EMT phenotype.

As ACSL3 seems to enhance the carcinogenic properties of NSCLC cells, we next evaluated the role of ACSL3 on cell bioenergetics, by monitoring the effect on the two main bioenergetic pathways: aerobic glycolysis and the mitochondrial oxidative phosphorylation. By mean of the Extracellular Flux Bioanalyzer (Seahorse Bioscience), we monitored (a) the extracellular acidification rate (ECAR) as an indirect readout of the lactate production by the aerobic glycolysis, and the (b) the oxygen consumption rate (OCR) as a readout of the oxidative phosphorylation at mitochondria (Materials and methods). Highly proliferative tumor cells frequently upregulate aerobic glycolysis (Warburg effect); nevertheless, we did not observed differences in the glycolytic performance of ACSL3 CRL 5908 compared to their control cells (data not shown).

As ACSL3 is a metabolic enzyme implicated in the activation of FAs, which is required prior to FAs entering into the β‐oxidation pathway at mitochondria, we monitored the oxidative phosphorylation at mitochondria by mean of the quantification of the OCR after injection of several modulators of the electron transport chain. Remarkably, ACSL3 CRL 5803 cells displayed increased basal and maximal respiratory capacities (increased spare respiratory capacity in stressed conditions) compared to the control No ORF CRL 5803 cells (Fig 2G‐bioenergetic profile, 2F).These results indicate that ACSL3 augments the cell bioenergetic capacity of the cells mainly by the enhancing the mitochondrial oxidative phosphorylation pathway, which may be related to the role of ACSL3 on FA activation for β‐oxidation at mitochondria.

All these data confirm the carcinogenic role of ACSL3 overexpression by increasing cell growth, migration, and invasion. Moreover, our data propose that metabolic reprogramming is associated with an ACSL3 increase in cancer cells.

### 3.3 Statins in non‐small cell lung cancer treatment

Anticancer use of non‐oncologic drugs is an emerging area of research with promising results. Statins, that are lipid‐lowering drugs, have been previously reported to display several anticancer properties. However, their exact roles in cancer initiation and progression have not been clearly elucidated, as well as the proper conditions to be used.

**Fig. 3:**
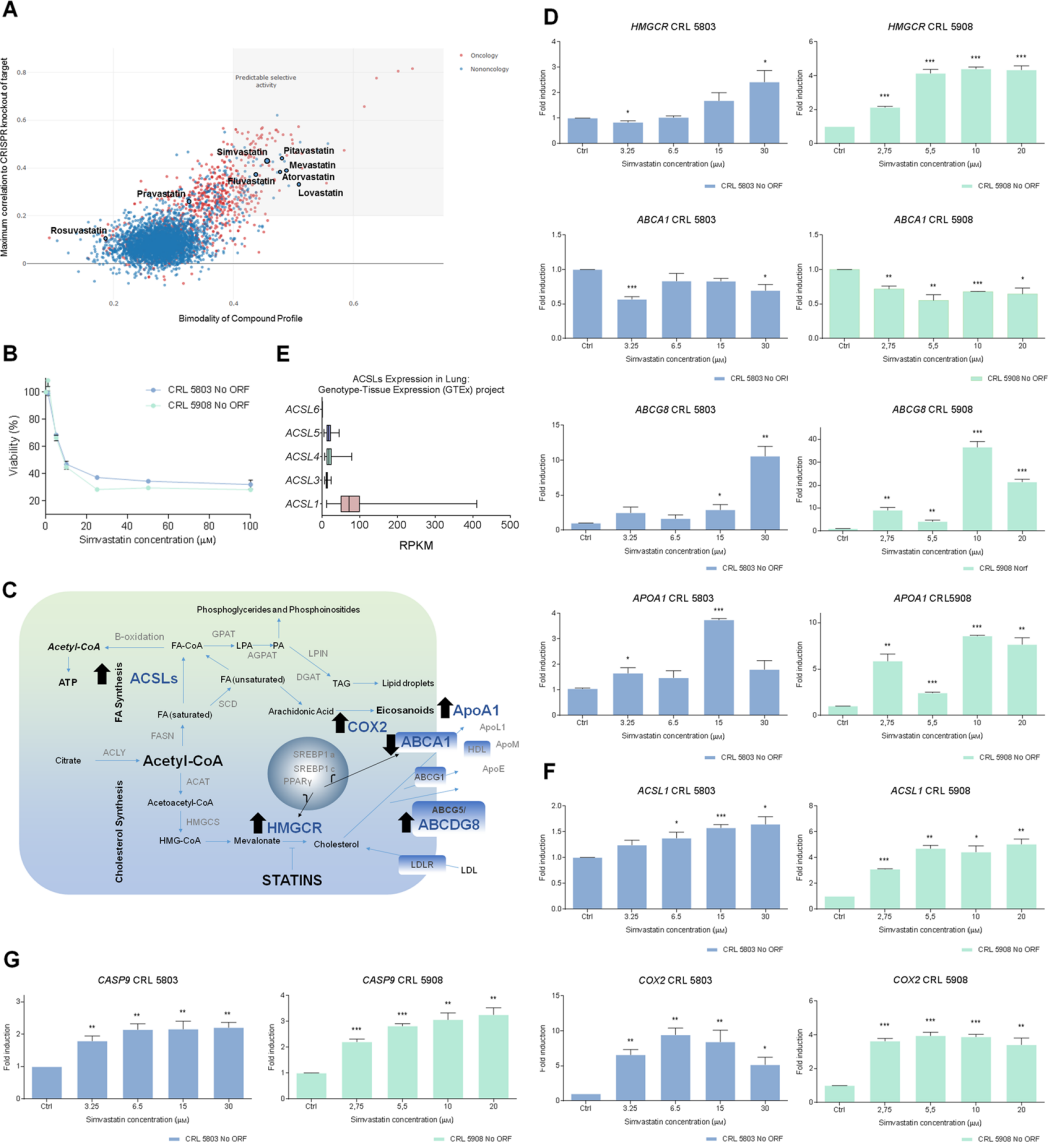
Simvastatin promotes antiproliferative effects and modulates lipid metabolism pathways in NSCLC cells. (A) Illustration adapted from Corsello *et al*. [44] using a public resource (https://depmap.org/repurposing/). We are showing statins in a graphic representing the relationship between the bimodality coefficient of the PRISM (drug repurposing resource viability) profile and correlation with the CRISPR knockout of the annotated gene target (oncology: *n* = 885 compounds; non‐oncology: *n* = 2361 compounds). Bimodality coefficients were calculated over cell lines shared by each compound and target shown. (B) Viability inhibition of NoORF CRL 5803 and CRL 5908 cells by simvastatin. Cancer cells were treated with increasing concentrations of simvastatin during 48 h, and cell viability was determined by MTT (3‐(4,5‐dimethylthiazol‐2‐yl)‐2,5‐diphenyltetrazolium) assay. Values are expressed as the mean ± SEM of at least three independent experiments, each performed in quadruplicate. (C) Schematic representation of changes of gene expression in cholesterol and fatty acids synthesis pathway after statins administration. (D) Gene expression in cholesterol synthesis pathway after statins administration. Relative expression assessed by means of qRT‐PCR of *HMGCR, ABCA1, ABCG8,* and *APOA1* genes of cholesterol synthesis pathway in NoORF CRL 5803 and CRL 5908 cells after 48 h of increasing concentrations of simvastatin treatments. Relative gene expression was calculated using 0 µm sample as control. Data correspond to mean ± SEM of three independent assays each performed in triplicate. Student’s t‐test was used to evaluate statistically significant differences (*P < 0.05, **P < 0.01, ***P < 0.001). (E) Acyl‐CoA synthetase isoforms abundance in non‐tumoral lung tissue. Expression data in RPKM (Reads per kilo base per million mapped reads) obtained from Genotype‐Tissue Expression (GTEx) project. (F) Gene expression in fatty acids synthesis pathway after statins administration. Relative expression assessed by means of qRT‐PCR of *ACSL1* and *COX2* genes of fatty acids synthesis pathway in NoORF CRL 5803 and CRL 5908 cells after 48 h of increasing concentrations of simvastatin treatments. Relative gene expression was calculated using 0 µm sample as control. Data correspond to mean ± SEM of three independent assays each performed in triplicate. Student’s t‐test was used to evaluate statistically significant differences (*P < 0.05, **P < 0.01, ***P < 0.001). (G) Gene expression in apoptosis after statins administration. Relative expression assessed by means of qRT‐PCR of *CASP9* in NoORF CRL 5803 and CRL 5908 cells after 48 h of increasing concentrations of simvastatin treatments. Relative gene expression was calculated using 0 µm sample as control. Data correspond to mean ± SEM of three independent assays each performed in triplicate. Student’s *t*‐test was used to evaluate statistically significant differences (*P < 0.05, **P < 0.01, ***P < 0.001).

We used a public resource (https://depmap.org/repurposing/), containing the growth‐inhibitory activity of 4518 drugs tested across 578 human cancer cell lines [44], to explore putative anticancer effects of statins. The most frequently used statin types have predicted selective activity in cancer cells. Statins selectively inhibited groups of cancer cell lines depending on their molecular features. Results are shown in Fig 3A.

With the aim of studying the anticancer effects of statins in human NSCLC cells, we determined the antiproliferative activity of simvastatin in our CRL 5803 and 5908 cell lines (Fig 3B). As a quantitative measure of this, we analyzed the IC_50_ parameter (concentration corresponding to 50% of cell viability inhibition) of simvastatin, showing similar sensitivity (IC_50_: 15.1 ± 1.59 µm in CRL 5803 and IC_50_: 9.3 ± 1.8 µm in CRL 5908).

Next, we examined the regulation of a selected panel of metabolic genes in the presence of growing concentrations of simvastatin. Nontreated cells were kept as reference controls. We measured genes related to apoptosis (*CASP9*) as well as genes implicated in *de novo* synthesis of cholesterol and FAs, genes regulating the intracellular lipid homeostasis—FA‐activating genes, lipid droplet formation, FA oxidation, extracellular lipid, and cholesterol uptake—and plasma membrane remodeling enzymes, among others (Fig 3C).

As expected, simvastatin treatment induced several transcriptional changes in the cholesterol biosynthetic pathway. We detected a consistent upregulation of *HMGCR* gene, *ABCG8* transporter, and apolipoprotein A1 (*APOA1*) together with a slight downregulation of the expression of the *ABCA1* cholesterol reverse transporter. These changes were observed in both NSCLC cell lines (Fig 3D).

The acyl‐CoA synthetase isoform 1 (ACSL1) is the most frequent isoform found in the nontumoral lung tissue, as it is shown in Fig 3E from Genotype‐Tissue Expression (GTEx) project. Simvastatin significantly modulated genes related to lipid metabolism, increasing the expression of *ACSL1* FA‐activating enzyme and *COX2* expression implicated in the synthesis of prostaglandins E2 (PGE2) (Fig 3F).

Finally, we observed a robust induction of *CASP9* expression in both cell lines (Fig 3G), indicating that simvastatin treatment induces apoptosis activation, as previously reported.

These data point out that simvastatin exerts an antitumoral effect modulating lipid metabolism and apoptosis in lung cancer tissue (Fig 3C).

### 3.4 Fatty acids metabolism through ACSL3 enzyme potentiates the antiproliferative effects of statins in NSCLC cells

Since fatty acid‐related *ACSL3* determines poor clinical prognosis in NSCLC, we analyzed the effect of statins administration in an *ACSL3* overexpressing scenario, which would correspond to 76.6% of the NSCLC patients.

**Fig. 4:**
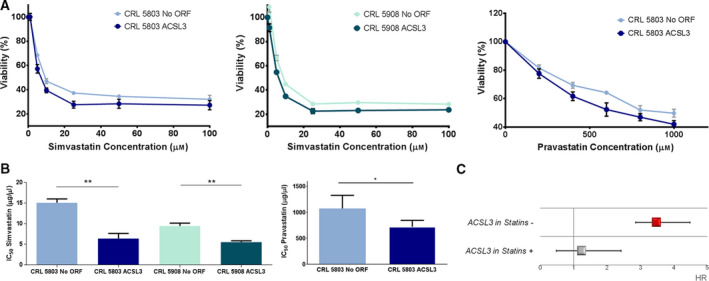
ACSL3 potentiates the antiproliferative effects of statins associated with better clinical outcome. (A) Viability inhibition of NoORF and ACSL3 CRL 5803 and CRL 5908 cells by statins. Cells were treated with increasing concentrations of simvastatin or pravastatin during 48 h, and cell viability was determined by MTT (3‐(4,5‐dimethylthiazol‐2‐yl)‐2,5‐diphenyltetrazolium) assay. Values are expressed as the mean ± SEM of at least three independent experiments, each performed in quadruplicate. (B) Bars represent the IC50 values (50% cell viability inhibition) determined in MTT assay, of the statins in each cancer cell line. Values are expressed as the mean ± SEM of at least three independent experiments, each performed in quadruplicate. Student’s *t*‐test was used to evaluate statistically significant differences (*P < 0.05, **P < 0.01). (C) Forest plot showing the hazard ratio (HR) and 95% confidence interval (CI) estimates for overall survival (OS) of *ACSL3* expression in 90 non‐small cell lung cancer (NSCLC) patients from Initial set according to their statins treatment.

As it is shown in Fig 4A, simvastatin reduced the IC_50_ value in ACSL3 CRL 5803 cells up to 57.4% compared to that observed in No ORF CRL 5803 cells (No ORF IC_50_: 15.1 ± 1.59 vs ACSL3 IC_50_: 6.4 ± 2.1). Similarly, simvastatin also reduced the IC_50_ value (up to 39%) in ACSL3 CRL 5908 compared to that found in No ORF CRL 5908 cells (NoORF IC_50_: 9.3 ± 1.8 vs ACSL3 IC_50_: 5.7 ± 0.7). Besides, these results were also confirmed after pravastatin treatment where the IC_50_ in ACSL3 CRL 5803 cells were reduced up to 32.9% compared to the IC_50_ determined for No ORF 5803 cells (NoORF IC50: 1076.1 ± 253 vs ACSL3 IC50: 721.7 ± 125) (Fig 4B). These results indicate that overexpression of ACSL3 augments the sensitivity to statins (lower IC_50_ values compared to control cells).

### 3.5 Redefining the role of statins as anticancer agents in NSCLC: precision medicine approaches based on ACSL3 lipid metabolism enzyme

Finally, we explored the association between statin use and cancer prognosis in NSCLC patients. As expected from previous reports, we were able to detect a trend between statin administration and survival in our set of high‐grade NSCLC patients. Thus, patients, who had followed a standardized statin treatment, tend to have better clinical outcome with a HR of 0.77 (95% CI: 0.45–1.33, *P* = 0.3) (see Table 1).

Importantly, the risk of death associated with *ACSL3* overexpression increased up to 3.47 (95% CI: 1.57–7.69, *P* = 5.36 × 10^−4^) in the 66 patients not following statin treatment vs those patients following a standardized statin treatment (*n* = 24), in which the expression of *ACSL3* was not a marker of risk (HR: 1.23 (95% CI: 0.35–4.3), *P* = 0.75) (Fig 4C). In our population, there are a 22.22% of patients that overexpress *ACSL3* and are having benefits from statin treatment. By contrast, there are a 57.77% of patients that overexpress *ACSL3* and would advantage from statin antiproliferative action. Thereby, these results suggest that *ACSL3* expression determines statins antitumoral activity and could be useful to select a potential candidate population of NSCLC patients that would significantly benefit from statin administration.

## 4 Discussion

Non‐small cell lung cancer remains among the most lethal cancers, despite the numerous scientific efforts made in last decade in diagnosis and treatment of the disease [45]. Global NSCLC mortality continues increasing, and molecular biomarkers of diagnosis and prognosis are still needed. Nowadays, promising evidences of putative benefits of precision medicine allow us an opportunity to fight against these types of tumors. Precision medicine will converge in the identification of specific groups of patients with particular characteristics and consequently with personalized treatments [46].

One of the hallmarks of cancer is metabolic reprogramming [8]. Cancer cells modify their metabolic capacities and reprogram their energy sources; subsequently, this reprogramming offers us a putative therapeutic opportunity. Furthermore, lipid metabolism has been raised as a potential target for cancer diagnosis, prognosis, and treatment [9]. It has been shown that lipid metabolism alterations are interesting biomarkers of cancer prognosis in several types of tumors [10, 11].

In the present study, we show that the expression of a lipid metabolism enzyme, ACSL3, classifies a group of NSCLC patients with poor survival, probably related to increased invasive properties moreover than proliferation‐related events, as previously shown of the involvement of lipid metabolism in cancer progression. Importantly, *ACSL3* expression is also categorizing patients who could benefit of putative antitumor properties of statin administration.

ACSL3 belongs to the family of long‐chain acyl‐CoA synthetases that is composed by five human isoforms (ACSL1, ACSL3, ACSL4, ACSL5, and ACSL6). They are lipid metabolic enzymes which convert free fatty acid to fatty acid‐CoA. Based on the sequence homology, ACSL isoforms are separated into two groups: One is composed of ACSL1, ACSL5, and ACSL6 and the other ACSL3 and ACSL4 [17]. They have different subcellular locations and substrate preferences but all of them converge in the fatty acid synthesis pathway. ACSL3 prefers substrates like myristate, palmitate, arachidonate, and eicosapentaenoate [16] and localizes to the periphery of the lipid droplets of lipogenic cells, and it is also present on the cytoplasmic face of the endoplasmatic reticulum [47].

Long‐chain acyl‐CoA synthetases have been proposed as putative prognostic biomarkers of cancer. *ACSL1* and *ACSL4* overexpression was associated with a poor clinical outcome in stage II colorectal cancer patients [10, 11, 18, 48, 49]. In addition, *ACSL4* is considered a biomarker for liver and breast cancer [50, 51]. By contrast, downregulation of *ACSL5* in breast cancer was associated with a worse prognosis [49, 52] and none study have been reported on the association of *ACSL6* and cancer survival [53]. An *in silico* study also suggested that high *ACSL1* expression was associated with worse survival in lung cancer patients, and *ACSL3* overexpression was associated with worse survival in patients with melanoma [49].

*ACSL3* was found to be overexpressed in prostate cancer [54] and estrogen receptor‐negative breast cancer [55]. Besides, tumor tissue microarray (TMA) staining [56] showed that ACSL3 is upregulated in lung cancer compared to the healthy lung tissue. However for the first time, in the present study, we report an association between *ACSL3* expression with the prognosis and survival in NSCLC. In our set of patients, high *ACSL3* expression levels showed an increased risk of death with a HR of 2.93 (95% CI: 1.5–5.73), *P* = 0.011 (Fig 1A). Moreover, we were able to replicate our results in a large sample of 984 NSCLC patients (HR: 1.38 (95% CI: 1.07–1.79), *P* = 0.016, Fig 1B). Importantly, these results are independent of putative confounding factors such as age, sex, stage, ECOG, histology, and smoking status and *P*‐values are corrected for multiple testing.

The poor survival of patients who exhibit high *ACSL3* expression levels is probably due to an increase in the carcinogenic capacities of the ACSL3 overexpressing cells. In our study, ACSL3 overexpression increased proliferation, migration, and invasion of tumor cells, which favors malignancy. ACSL3 overexpression increased cell growth in CRL 5803 cells but not in CRL 5908 cells. We hypothesized that the specific cellular background probably explains these differences. Nevertheless, migration and invasion properties are significantly increased in both cellular types upon ACSL3 overexpression (Fig 2C–F). Moreover, high expression levels of ACSL3 also augmented the mitochondrial oxidative capacity (increased basal and maximal respiratory capacity) (Fig 2G,H). It is tempting to speculate that ACSL3, due to its function on the activation of FAs, may contribute to augment the FA β‐oxidation capacity of the cells conferring an advantage to tumor cells.

These results are in line with the observations that the ACSLs are upregulated in cancer. It has been proposed that ACSL‐mediated lipid anabolism may support cancer initiation. Fatty acids promote tumor cell proliferation by providing crucial biosynthetic and functional intermediates. Independent knockdown of *ACSL1*, *ACSL3,* or *ACSL4* decreased cell proliferation and anchorage‐independent growth in many cancer cells and xenograft tumor growth in nude mice [50, 53, 56, 57, 58]. By contrast, overexpression of these ACSLs increased cell proliferation and tumor growth [51, 59, 60, 61, 62]. Also, ACSL1 and ACSL4 overexpression increases migration and invasion in colon cancer cells [18] but not proliferation and contribute to a non‐Warburg advantageous energetic status of invasive colon cancer cells [57].

All these data allow us to hypothesize that, in NSCLC, ACSL3 overexpression is defining a metabolic scenario, associated with increased fatty acids activation conducting to an aggressive and invasive tumor cell and consequently with poor patient survival.

Statins are able to modulate the metabolic context since they are cholesterol‐lowering drugs. They act by competitively inhibiting 3‐hydroxy‐3‐methylglutaryl‐coenzyme A (HMG‐CoA) reductase, decreasing the endogenous cholesterol synthesis. Emerging evidences suggest that statins display antitumoral, apoptotic, and immunomodulatory properties [21, 27].

Statins have been shown to potentially decrease cancer risk and to improve survival in patients. Their anticancer effects have been described in several types of cancer, and several epidemiological and clinical studies have investigated the role of statins in protecting against lung cancer. Although much debate has been focused in describing their effects, it seems that high stage NSCLC patients could benefit of the statin administration [63, 64].

The precise molecular mechanism by which statins generate anticancer effects is unknown, although appear to include inhibition of the mevalonate/cholesterol synthesis pathway, thus blocking the synthesis of intermediates key for prenylation and activation of the Ras/mitogen‐activated protein kinase 1 signaling pathway. Moreover, statins have been recognized to modulate the phosphoinositide 3‐kinase/Akt serine/threonine kinase 1 and inflammation signaling pathways and to modify lipid metabolism gene expression [27, 65].

In the present study, we demonstrated that simvastatin exerts an antiproliferative action in NSCLC cell lines followed by transcriptional changes in lipid metabolism genes: *HMGCR, ABCG8, APOA1, ABCA1, ACSL1,* and *COX2* (Fig 3B,C,D,G,F). ACLS1 isoform is the most abundant one in the healthy lung tissue (Fig 3E); consequently, it is probably that *ACSL1* transcription was the most finely regulated among ACSLs isoforms. We also demonstrated that simvastatin induces apoptosis in NSCLC cell lines *via* induction of *CASP9* (Fig 3G).

Recent studies are exploring the use of common drugs for cancer therapy [44]. Based on them, we proposed that statins could be use in selective tumor scenarios (Fig 3A). Here, we are tagging one of these scenarios; our data indicate that an ACSL3‐upregulated context enlarges the antiproliferative effect of statins treatment in NSCLC cells (Fig 4A,B). In addition to *ACSL3*, it could be some other genes implicated in the mechanisms of response of tumor cells to statins.

In our study, and accordingly to recent reported results, we detected a trend between statin administration and better prognosis in NSCLC patients (Table 1). However, we found that significantly *ACSL3* overexpression increases risk of death with HR of 3.47 (95% CI: 1.57–7.69, *P* = 5.36 × 10^−4^) in patients not following statin treatment, while in those patients following statin treatment, *ACSL3* is not a marker of risk (Fig 4C).

Thereby, though a prospective clinical study would be necessary to further establish the clinical benefit of *ACSL3* determination as a biomarker of statins benefit, these results strongly suggest that those patients overexpressing *ACSL3* will obtain a clinical benefit from statin administration. The attractiveness of statins as part of chemotherapeutic drug arsenal lies in their relatively safe and well‐tolerated toxicity profile together with their low cost. Thus, a metabolic biomarker of worse clinical outcome of NSCLC patients, together with a targeted well‐tolerated treatment to overcome its aggressive potential, is the first time reported here.

## 5 Conclusions

A crucial point on cancer management is to diagnose patients who would benefit from specific therapies. Here, we found that the overexpression of a fatty acid‐related ACSL3 enzyme could be considered as an unfavorable prognostic marker in non‐small cell lung cancer (NSCLC). Moreover, the overexpression of *ACSL3* increased cell proliferation, migration, and invasion altering metabolic properties of lung cancer cells.

We found that statin administration caused antiproliferative effects, and importantly, those patients with high expression levels of *ACSL3* following statins treatment displayed significant better prognosis than those without statins treatment.

ACSL3 emerges as a biomarker for clinical prognosis in NSCLC for the identification of patients that would benefit from anticancer properties of statin treatments that are safe, well‐tolerated, and low cost.

## Conflict of interest

The authors declare no conflict of interest.

## Author contributions

LPF, MM, MS, and ARM conceptualized and designed the study. LPF, JMR, RSM, AQF, and MGC involved in development of methodology. LPF, MM, JMR, RSM, MS, GR, and EC acquired the data. LPF, MM, EC, MS, and ARM analyzed the interpreted the data. LPF and GC involved in survival analysis. LPF, GC, MGC, and ARM wrote and reviewed the manuscript.

## For more information

The Cancer Genome Atlas (TCGA): https://www.cancer.gov/about‐nci/organization/ccg/research/structural‐genomics/tcga. The Human Protein Atlas: https://www.proteinatlas.org/. The Pathology Atlas: https://www.proteinatlas.org/humanproteome/pathology. Cancer Cell Line Encyclopedia: https://portals.broadinstitute.org/ccle. COSMIC database: https://cancer.sanger.ac.uk/cosmic.

## Supporting information


**Fig S1.** Distributions of*ACSL3*, *NID1* and *RETN* gene expression. Violin plots displaying distributions of *ACSL3*, *NID1* and *RETN* gene expression in 90 NSCLC patients. The horizontal segment indicates the cutpoint used in their binarization.

**Fig S2.** Generation of ACSL3 cellular models. A. mRNA expression levels of *ACSL3* measured by RT‐QPCR, in non‐infected NSCLC cells and HEK293 cells. Data represent mean ± SEM of three independent experiments. B. *ACSL3* expression levels extracted from the Cancer Cell Line Encyclopedia database (https://portals.broadinstitute.org/ccle/about). C. *ACSL3* expression levels from COSMIC (https://cancer.sanger.ac.uk/cosmic) database. D. mRNA expression levels of *ACSL3* assayed by RT‐QPCR, in stable cell lines overexpressing ACSL3. Data correspond to mean ± SEM of three independent assays. Student’s t test was used to evaluate statistically significant differences (*P < 0.05, ***P < 0.001). E. Protein expression levels of ACSL3 in non‐infected NSCLC cells and HEK293 cells transient transfected with NoORF and ACSL3 vectors Proteins were detected by western blot using specific antibodies against ACSL3 and β‐Actin, as a loading control. F. Protein expression levels of ACSL3 in stable cell lines overexpressing ACSL3. Proteins were detected by western blot using specific antibodies against ACSL3 and β‐Actin, as a loading control.

**Fig S3.** Epithelial–mesenchymal transition (EMT) Markers in ACSL3 NSCLC cells. A. mRNA expression levels of several EMT markers measured by RT‐QPCR, in No ORF and ACSL3 CRL 5803 cells. Data represent mean ± SEM of three independent experiments. B. mRNA expression levels of several EMT markers measured by RT‐QPCR, in No ORF and ACSL3 CRL 5908 cells. Data represent mean ± SEM of three independent experiments.

**Table S1.** Clinical characteristics of non‐small cell lung cancer patients from The Cancer Genome Atlas (TCGA) external validation set.

**Table S2.** List of oligos used in this study.
