# Supplementary figures and images for: Metabolic enzyme ACSL3 is a prognostic biomarker and correlates with anticancer effectiveness of statins in non‐small cell lung cancer
Source: Mol Oncol. 2020 Oct 30;14(12):3135–52. doi: 10.1002/1878-0261.12816 (PMC7718959; doi:10.1002/1878-0261.12816)

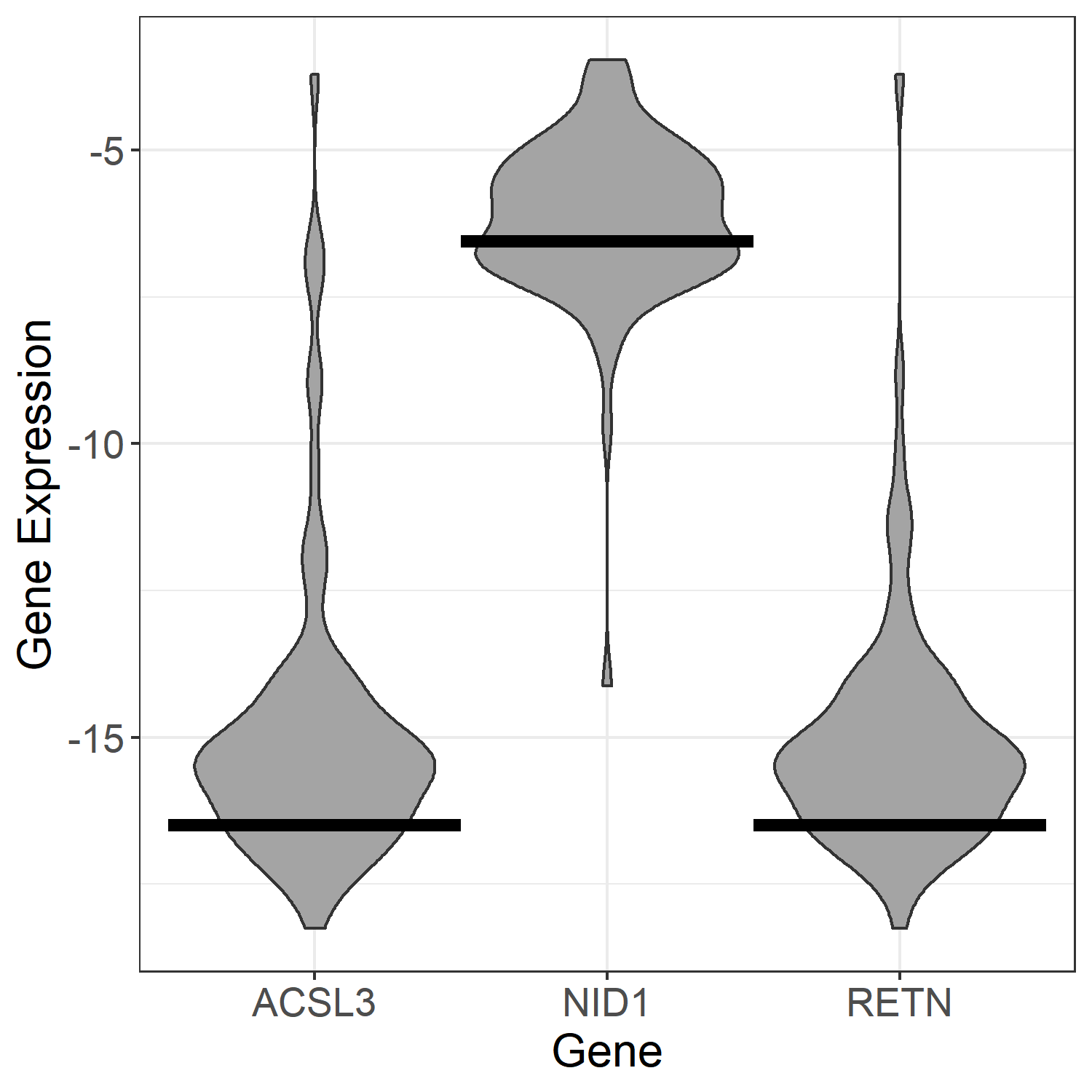

Supplement: Supplementary file 1 — Fig S1. Distributions ofACSL3, NID1 and RETN gene expression. Violin plots displaying distributions of ACSL3, NID1 and RETN gene expression in 90 NSCLC patients. The horizontal segment indicates the cutpoint used in their binarization. [file MOL2-14-3135-s001.tif]

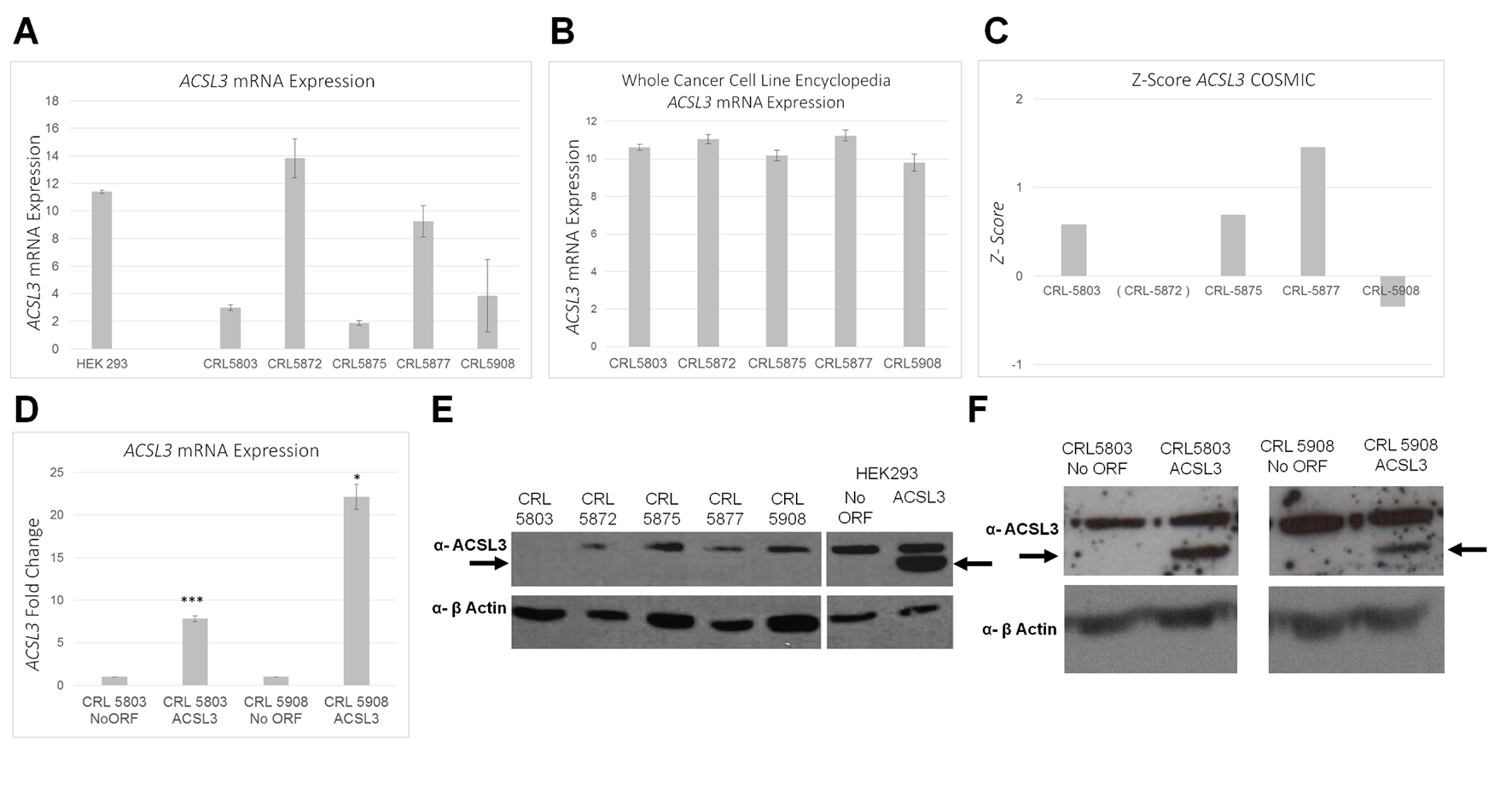

Supplement: Supplementary file 2 — Fig S2. Generation of ACSL3 cellular models. A. mRNA expression levels of ACSL3 measured by RT‐QPCR, in non‐infected NSCLC cells and HEK293 cells. Data represent mean ± SEM of three independent experiments. B. ACSL3 expression levels extracted from the Cancer Cell Line Encyclopedia database (https://portals.broadinstitute.org/ccle/about). C. ACSL3 expression levels from COSMIC (https://cancer.sanger.ac.uk/cosmic) database. D. mRNA expression levels of ACSL3 assayed by RT‐QPCR, in stable cell lines overexpressing ACSL3. Data correspond to mean ± SEM of three independent assays. Student’s t test was used to evaluate statistically significant differences (*P < 0.05, ***P < 0.001). E. Protein expression levels of ACSL3 in non‐infected NSCLC cells and HEK293 cells transient transfected with NoORF and ACSL3 vectors Proteins were detected by western blot using specific antibodies against ACSL3 and β‐Actin, as a loading control. F. Protein expression levels of ACSL3 in stable cell lines overexpressing ACSL3. Proteins were detected by western blot using specific antibodies against ACSL3 and β‐Actin, as a loading control. [file MOL2-14-3135-s002.tif]

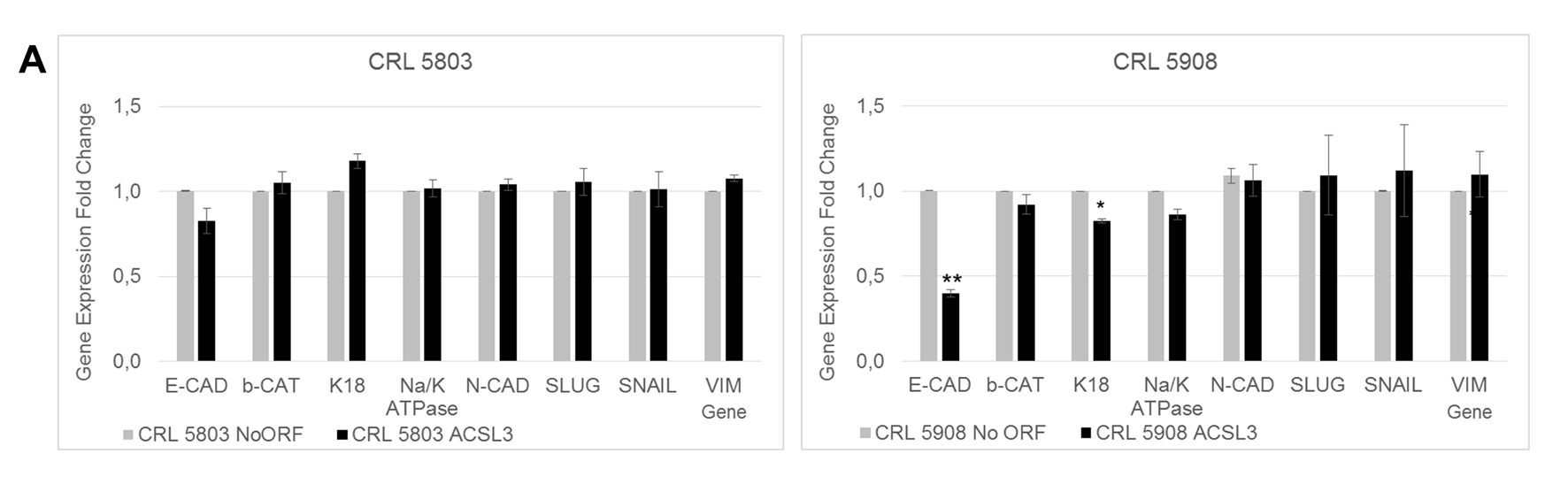

Supplement: Supplementary file 3 — Fig S3. Epithelial–mesenchymal transition (EMT) Markers in ACSL3 NSCLC cells. A. mRNA expression levels of several EMT markers measured by RT‐QPCR, in No ORF and ACSL3 CRL 5803 cells. Data represent mean ± SEM of three independent experiments. B. mRNA expression levels of several EMT markers measured by RT‐QPCR, in No ORF and ACSL3 CRL 5908 cells. Data represent mean ± SEM of three independent experiments. [file MOL2-14-3135-s003.tif]
