# Supplementary material for: Metabolic enzyme ACSL3 is a prognostic biomarker and correlates with anticancer effectiveness of statins in non‐small cell lung cancer
Source: Mol Oncol. 2020 Oct 30;14(12):3135–52. doi: 10.1002/1878-0261.12816 (PMC7718959; doi:10.1002/1878-0261.12816)
Supplement: Supplementary file 4 — Table S1. Clinical characteristics of non‐small cell lung cancer patients from The Cancer Genome Atlas (TCGA) external validation set. [file MOL2-14-3135-s004.pdf]

Table S1

		N(%)
**Number of patients**		984(100)
**Number of exitus**		274 (27.85)
**Age**		
Mean		66.27
Median		67
Range		38-90
<50		49 (4.98)
50-70		583 (59.25)
>70		352 (35.77)
**Sex**		
Male		590 (59.96)
Female		394 (40.04)
**Histology**		
Adenocarcinoma		495 (50.3)
Squamous		489 (49.7)
**Stage**		
	1	502 (51.65)
	2	274 (28.19)
	3	163 (16.77)
	4	33 (3.4)
