# Supplementary material for: Metabolic enzyme ACSL3 is a prognostic biomarker and correlates with anticancer effectiveness of statins in non‐small cell lung cancer
Source: Mol Oncol. 2020 Oct 30;14(12):3135–52. doi: 10.1002/1878-0261.12816 (PMC7718959; doi:10.1002/1878-0261.12816)
Supplement: Supplementary file 5 — Table S2. List of oligos used in this study. [file MOL2-14-3135-s005.pdf]

Table S2

Gene	Orientation	Sequence (5'->3')
ACSL3	Forward	GGCGTAGCGGTTTTGACAC
	Reverse	CCAGTCCTTCCCAACAACGA
E-CAD (CDH1)	Forward	GAACGCATTGCCACATACAC
	Reverse	GAATTCGGGCTTGTTGTCAT
K18 (KRT18)	Forward	GAGTATGAGGCCCTGCTGAA
	Reverse	CAGACACCACTTTGCCATCC
Na/K ATPase (ATP1B1)	Forward	GCCTCCCAAGAATGAGTCCT
	Reverse	ATTTGGGCTGCAGGAGTTTG
N-CAD (CDH2)	Forward	CGGTTTCATTTGAGGGCACA
	Reverse	TTGGAGCCTGAGACACGATT
SLUG (SNAI2)	Forward	CGTTTTCCAGACCCTGGTT
	Reverse	CTGCAGATGAGCCCTCAGA
VIM	Forward	GAGTCCACTGAGTACCGGAG
	Reverse	ACGAGCCATTTCCTCCTTCA
HMGCR	Forward	TGATTGACCTTTCCAGAGCAAG
	Reverse	CTAAAATTGCCATTCCACGAGC
ABCA1	Forward	ACCCACCCTATGAACAACATGA
	Reverse	GAGTCGGGTAACGGAAACAGG
ABCG8	Forward	AGCCTCCTTGCTAGATGTGAT
	Reverse	GTCTCTCGCACAGTCAAGTTG
APOA1	Forward	CCCTGGGATCGAGTGAAGGA
	Reverse	CTGGGACACATAGTCTCTGCC
ACSL1	Forward	ACATTATGTTCCTGGGCCCA
	Reverse	AGTCAGAAGGCCATTGTCGA
COX2	Forward	ATCACAGGCTTCCATTGACC
	Reverse	CAGGATACAGCTCCACAGCA
CASP9	Forward	CAGGCCCCATATGATCGAGG
	Reverse	TCGACAACTTTGCTGCTTGC
GAPDH	Forward	TGGTATCGTGGAAGGACTCATGAC
	Reverse	ATGCCAGTGAGCTTCCCGTTCAGC
B2M	Forward	GATGAGTATGCCTGCCGTGT
	Reverse	TGCGGCATCTTCAAACCTCC
